# A theoretical study of benzaldehyde derivatives as tyrosinase inhibitors using Ab initio calculated NQCC parameters

**Published:** 2015-09

**Authors:** Marjan Rafiee, Masoumeh Javaheri

**Affiliations:** 1 Department of Chemistry, Payame Noor University, Tehran, Iran; 2 Department of Ceramic, Materials and Energy Research Center, Karaj, Iran

**Keywords:** Charge density, NQR, Gaussian, quadrupolar nuclei

## Abstract

Tyrosinase is a multifunctional copper-containing enzyme. It can catalyze two distinct reactions of melanin synthesis and benzaldehyde derivatives, which are potential tyrosinase inhibitors. To find the relationships between charge distributions of benzaldehyde and their pharmaceutical behavior, the present study aimed at investigating nuclear quadrupole coupling constants of quadrupolare nuclei in the functional benzaldehyde group and calculating some its derivatives. In addition, the differences between the electronic structures of various derivatives of this depigmenting drug were examined. All ab initio calculations were carried out using Gaussian 03. The results predicted benzaldehyde derivatives to be bicentral inhibitors; nevertheless, the oxygen or hydrogen contents of the aldehyde group were not found to be the only active sites. Furthermore with the presence of the aldehyde group, the terminal methoxy group in C4 was found to contribute to tyrosinase inhibitory activities. In addition, an oxygen atom with high charge density in the side chain was found to play an important role in its inhibitory effect.

## INTRODUCTION

Human skin pigmentation occurs as a result of the accumulation of melanin in the epidermis, which is produced by melanocytes within specialized organelles called melanosomes. Tyrosinase (EC. 1.14.18.1) is a copper-containing enzyme widely distributed in fungi, higher plants, and animals. This enzyme catalyzes two key reactions. The melanin biosynthesis pathway, which is the hydroxylation of monophenol to o-diphenol (monophenolase activity) and the conversion of an o-ygen species (ROS) [1, 2].

Tyrosinase inhibitors have become increasingly important in medication [3] and cosmetic [4] and food industries due to their role in decreasing the excessive accumulation of pigmentation resulting from enzyme activities [5-9]. Previous reports have confirmed tyrosinase to be effective in animal melanizing as well as plant browning. Recent investigations have demonstrated various dermotological disorders, such as age spots and freckles to be caused by the accumulation of excessive levels of epidermal pigmentation [10].

So far, numerous benzaldehyde derivatives and their analogues as potential tyrosinase inhibitors have been discovered from natural or synthetic sources, such as 4- hydroxybenzaldehyde [3], anisaldehyde [3], cuminaldehyde [11] and 4-methoxysalicyl- aldehyde [12]. Unfortunately, most 4-substituted benzaldehyde derivatives cannot be considered for practical use because of their lower activity levels and/or serious side effects. Therefore, the search for and discovery of novel tyrosinase inhibitors with higher activities and lower side effects is necessary. Nuclear quadrupole resonance (NQR) spectroscopy [13] is a very sensitive technique for measuring electric charge distributions around quadrupolar nuclei (I > 1/2).

Since NQR parameters are very sensitive to electronic structures, calculating them can reveal details of nuclei charge distributions (such as atomic charge determination) which may not be observed by other methods. The quantum mechanical approach has been shown to be an effective method of determining the charge distribution of molecules or complexes [14]. In this method, the electric field gradient (EFG), resulting from whole molecular charges, can be estimated at any point in the molecular space [15]. Verification of the EFG has become possible by quadrupolar nuclei which possess nuclear quadrupole moments and interact with molecular EFG tensors [16]. This interaction is measured by the nuclear quadrupole coupling constant (NQCC).

Even though experiments can provide information regarding the phenomenon, theoretical modeling has become crucial due to the fact that it can allow researchers to find out why it is happening and what will happen in similar conditions. The purpose of this study was to find the relationship between the electronic structure of benzaldehyde derivatives and their tyrosinase inhibitory ability using NQR parameters, due to the fact that these parameters are highly sensitive to local charge distribution.

## MATERIALS AND METHODS

Quantum mechanical calculations were performed using the Gaussian 03 program [17]. All structures were fully optimized without any restriction using the B3LYP functional. Through calculating vibrational frequencies, the optimized structures were characterized as minima (NIMAG=0). The B3LYP functional combines Becke's three- parameter exchange functional [18] with the correlation functional of Lee, Yang and Parr [19, 20].

In addition, since there is no experimental data on the NQCCs of the considered compounds, a rather small basis set such as 6-31G* can lead us to qualitative results using calculated NQCCs. This seems reasonable since a qualitative prediction may be obtained faster. In this work, ab initio calculations were performed at the B3LYP/6-31G* level to optimize and compute the components of the electric field gradient (EFG)tensor in the principal axes system.

**Evaluations of NQCCs**: The formulation employed in the evaluation of NQR parameters can be found in Graybeal (1988) [15]. Briefly, the EFG is a traceless, symmetric second-rank tensor in which principal axes are chosen so that its components satisfy $\left|q_{\mathrm{zz}}\right|\geq \left|q_{\mathrm{YY}}\right|\geq \left|q_{\mathrm{XX}}\right|,({\mathrm{eq}}_{\mathrm{ij}}=\frac{\partial^{2}V}{\partial_{i}\partial_{j}})$ where I, j=X, Y, Z *e* is electron charge and *V *is the external electronic potential [21]. The expression χ=e2 Qqzzh is termed as nuclear quadrupole coupling constant ( *NQCC=* *x* ) and has the unit of frequency (Hz), *h* is Planck’s constant, *Q *is nuclear electric quadrupole moment and *qzz *is the Z component of the EFG tensor in the principal axes system.

## RESULTS AND DISCUSSION

Similar to our previous studies [23, 25-26], in the present work ab initio NQCC computations were performed on a number benzaldehyde derivatives so that a possible relationship between their electronic structure and biological activity could be investigated.

Theoretical calculations, particularly those of nuclei NQCCs, seem to be proper tools for obtaining a better understanding of the electronic structure of these inhibitors.

**Figure 1:**
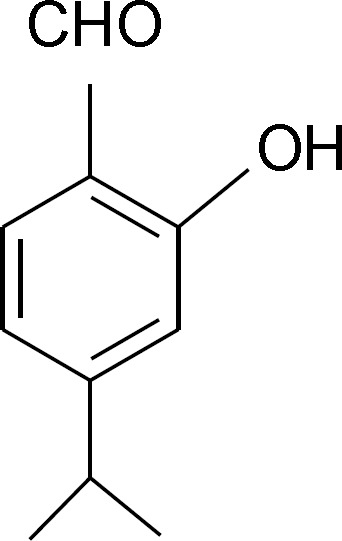
Structure of chamacein

**activities using calculated NQCCs: **At this stage, the NQCC of quadrupolare nuclei in the benzaldehyde functional group (B1) were calculated together with its derivatives (B2, B3, B4, B5 and B6; Table 1). These calculations were carried out to find relationships between the benzaldehyde charge distribution and its pharmaceutical behavior in addition to examining the differences between the electronic structures of various derivatives of this depigmenting drug. Nihei et al., recently studied the synthesis and tyrosinase inhibitory characteristics of chamaecin (2-hydroxy-4- isopropylbenzaldehyde) [12], whose structure is shown in Figure 1.

Kubo and Kinst-Hori (1999) isolated 2-hydroxy-4-methoxybenzaldehyde (B5) as a potent tyrosinase inhibitor from an African medicinal plant that inhibits the oxidation of L-DOPA catalyzed by mushroom tyrosinase with an IC50 of 30 μM. Later kinetic studies showed it to be a mixed-type inhibitor [27]. However, compound B6 (known as chamaecin) exhibited more potency compared to B1 with a similar type of reaction kinetics [27]. The calculated NQCCs of hydrogen and oxygen atoms in the aldehyde group of the above mentioned compounds are reported in Table 1.

**Table 1:** Comparison of quadrupolar atoms’ calculated NQCCs in measured derivatives of benzaldehyde

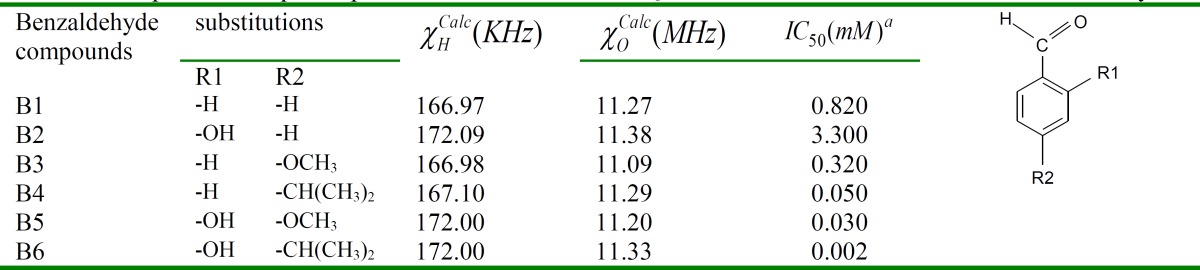

A close inspection of Table 1 shows that in B5 and B6 (compounds with an electron donating group at C-2 and C-4), hydrogen atoms have large NQCCs and therefore greater charge density. In addition, parallel to the greater charge density of hydrogen in these compounds, their oxygen atom charge density also increased. As Table 1 shows, the oxygen atom NQCC in B5 and B6 is smaller than that of other compounds such as B2, which is the least inhibitor in this group, by 180 KHz and 50 KHz, respectively. Since the contribution of nonbonding electrons (lone pairs p and d) in the nonspherical charge distribution is greater than the bonding electrons and charges of neighboring ions, atoms with nonbonding electron pairs (such as oxygen) have a these atoms causes nonbonding electron pairs to become modest, resulting in the increased symmetry of the EFG around the nucleus. As a result, oxygen atoms, decrease when their charge density increases. 

Based on calculated NQCC parameters, a two-center intermediate can be proposed as the mechanism of action of these inhibitors. In addition, charge densities of the aldehyde group oxygen and hydrogen atoms are understood to have a dominant role in the biological activity of benzaldehyde derivatives. When oxygen and hydrogen atoms in the aldehyde group have large charge densities, they prefer chelating with a cation such as Cu2+ over the others due to the fact that two Cu2+ ions are cofactors required for tyrozinase enzymatic activity. Such an assumption has been reinforced by their lower IC50; in a B2 compound that has the lowest inhibitory potency and the highest IC50, the oxygen atom has the lowest charge density and thus, the highest NQCC.

**Figure 2:**
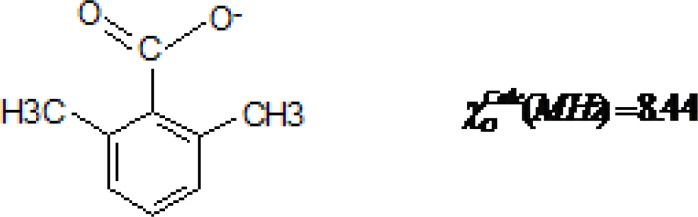
2,4 dimethylbenzoate (non inhibitor

When the aldehyde group at C-1 in the benzene ring is replaced by a carboxy group (figure 2, compound 2,4 dimethyl benzoate), it completely loses its inhibitory potency [28]. It can be thus concluded that benzaldehyde derivatives are bicentral inhibitors and oxygen or hydrogen alone are not active sites. This point can be seen in 2,4 dimethyl benzoate in which the charge density of the oxygen atom is much higher than that of the other compounds considered in figure 2 (the lowest NQCC; 8.44MHz), but whose inhibitory potency is vanished. In other words, the aldehyde group is necessary for the inhibitory treatment of the compound.

**Figure 3:**
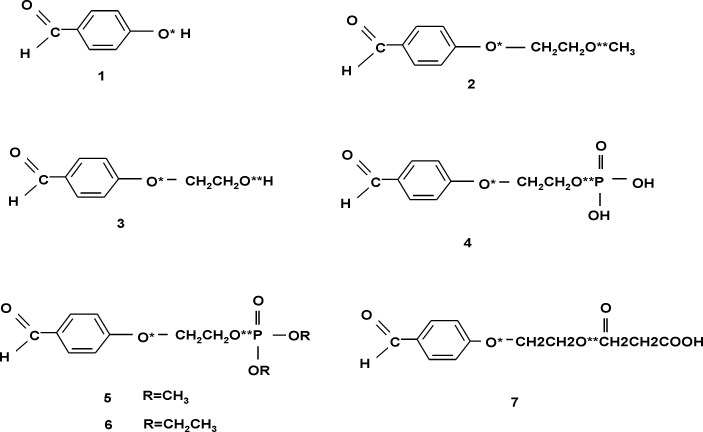
Selected derivatives of 4-hydroxybenzaldehyde

**Study of 4-hydroxybenzaldehyde derivatives and the effect of various side chains: **Tyrosinase is a multifunctional copper-containing enzyme. Tyrosinase is well known for its ability to catalyze two distinct melanin synthesis reactions. Nevertheless, it is still necessary to search and discover novel tyrosinase inhibitors with higher activities and lower side effects. Previous studies [3, 12] have reported the 4- substituted benzaldehyde derivatives to exhibit tyrosinase inhibitory activities by forming a Schiff base between the aldehyde group and a primary amino group of the enzyme. Other studies have maintained that the inductive effect of the electron-donating groups at position-4 is necessary for these actions [11]. Charge densities on O and H atoms are, therefore, expected to increase in order to form a better Schiff base with a primary amino group and to chelate with copper in the active site As a result, any status that increases the charge distribution of these atoms may cause an increase in the biological activity of 4- substituted benzaldehyde derivatives. Figure 3 presents the calculated NQCCs of quadrupolare nuclei in the 4-hydroxy benzaldehyde and some of its derivatives. These calculations were carried out to find relationships between the charge distribution of these compounds and their pharmaceutical behavior (Table 2).

**Table 2:** Comparison of calculated oxygen atom NQCCs in the 4-hydroxybenzaldehyde derivatives

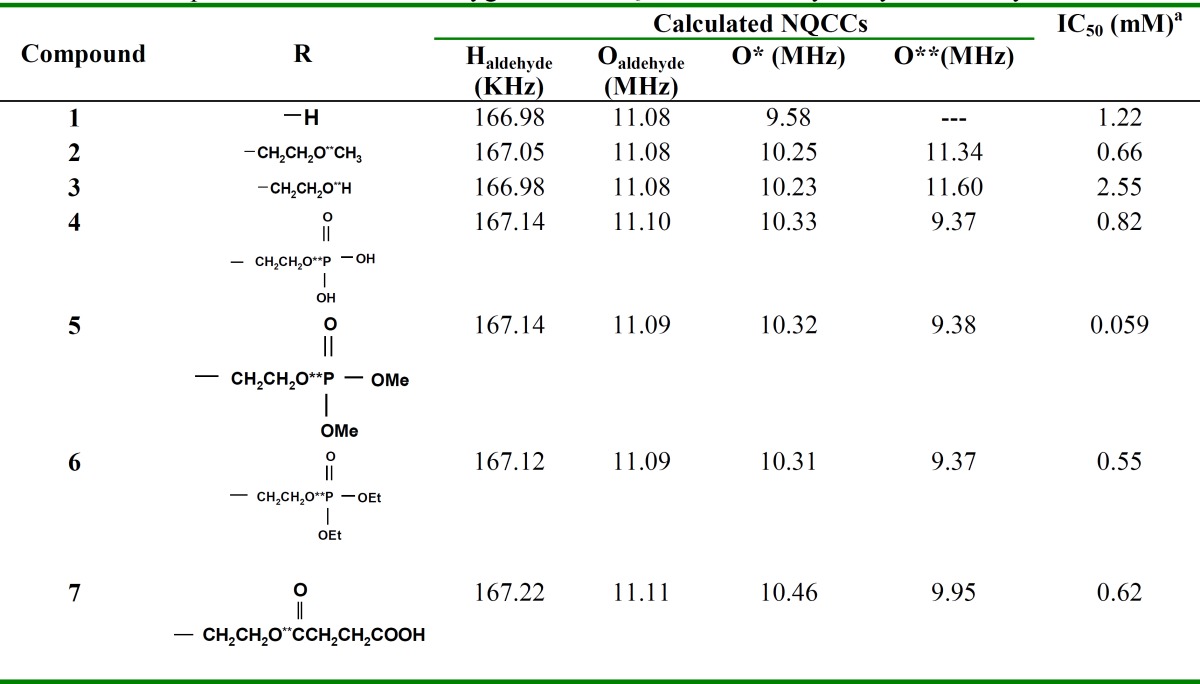

^a^ Experimental value from Ref. 29

Table 2 shows that in all the considered compounds, the aldehyde group hydrogen and oxygen have nearly equal NQCCs and thus, nearly equal charge densities. On the other hand, all compounds exhibited tyrosinase inhibitory effects with the IC50 ranging from 0.059 to 2.55 mM [29]. For example, bearing a hydroxyl ethoxyl group at position-4 of the phenyl ring, compound 3 exhibited a weak tyrosinase inhibitory activity with an IC50 value of 2.55 mM. Furthermore, compound 5 that bears adimethoxyl phosphate substituent is the most potent inhibitor with an IC50 value of 0.059 mM. Dictating the functional group charge density of benzaldehyde derivatives isnot the sole criteria for better inhibitory activity as the compounds' chemical structure seems to play an equally important role in determining their inhibitory strength.

The calculated oxygen and hydrogen NQCCs of the aldehyde group as well as the hydroxyl group oxygen atom (O*) and the side chain oxygen (O**) are reported. Table 2 shows that in compound **5**, which is the most potent inhibitor with an IC50 value of0.059 mM, NQCC of O** (oxygen in side chain) is the least. In other words, this oxygen has a larger charge density than any other compound.

In contrast, for compound **3 **that bears a hydroxyethoxyl group at position-4 of the phenyl ring, O**-NQCC is larger than the other compounds (11.60 MHz). This compound exhibited a weak tyrosinase inhibitory activity with an IC50 value of 2.55mM. The larger value of *x*(O**) in compound **3 **(about 2 MHz) compared to *x*(O**) in the other considered compounds indicates the smaller charge density of O** in thiscompound. This difference is an important criterion of the activity of 4-hydroxyl benzaldehyde derivatives suggesting that introducing appropriate hydrophobic subunits into position-4 of the benzaldehyde might facilitate their inhibitory effects. In other words, other than the aldehyde group, the terminal methoxy group may affect tyrosinase inhibitory activities. In addition, high charge density oxygen atoms in the side chain might also play an important role in determining their inhibitory effects on tyrosinase activities.

According to the data obtained from charge distributions, it can be concluded that:

- Quadrupolar parameters of nuclei can be used as useful tools to understand the electronic structure of compounds.

- The results imply that for B5 and B6, oxygen and hydrogen atoms of the aldehyde group have larger charge densities, and hence better enzyme-drug interaction and greater drug activity compared to other compounds. This is shown by experimental IC50 values; a smaller value for IC50 means greater activity.

- In the 4- hydroxyl benzaldehyde derivatives, as well as the aldehyde group, the terminal methoxy group may affect tyrosinase inhibitory activities and the charge density of the oxygen atom in the side chain might play an important role in determining their inhibitory effects on the tyrosinase activity.

## Notes

**Acknowledgements: **This research was financially supported by Payame NoorUniversity, Tehran.
